# Reemergence of Foot-and-Mouth Disease, South Korea, 2000–2011

**DOI:** 10.3201/eid2012.130518

**Published:** 2014-12

**Authors:** Jong-Hyeon Park, Kwang-Nyeong Lee, Su-Mi Kim, Hyang-Sim Lee, Young-Joon Ko, Dong-Seob Tark, Yeun-Kyung Shin, Min-Goo Seo, Byounghan Kim

**Affiliations:** Animal and Plant Quarantine Agency, Gyeonggi-do, South Korea

**Keywords:** foot-and-mouth disease, foot-and-mouth disease virus, viruses, outbreaks, reemergence, transmission, South Korea

## Abstract

Five outbreaks of foot-and-mouth disease have occurred in South Korea during 2000–2011. Macro-analysis of these outbreaks showed a correlation with outbreaks in countries in eastern Asia. Genetic analyses of food-and-mouth disease viruses in South Korea showed a correlation with viruses that are prevalent in neighboring countries.

Foot-and-mouth disease is an infectious viral disease that occurs in animals and is easily transmissible. Outbreaks of this disease affect international trade (*1*). Since 2000, five outbreaks (in March 2000, May 2002, January 2010, April 2010, and November 2010–April 2011) have occurred in South Korea; the outbreak in 2000 was the first in 66 years (*2*–*8*).

To better understand the risks associated with reemergence of this disease in South Korea, we examined characteristics of these outbreaks and those occurring in neighboring countries by using macroscopic analysis. We describe the outbreak patterns to enable prediction and prevention of this disease in South Korea.

## The Study

Spatiotemporal analyses used data obtained from the World Organisation for Animal Health Information Database (http://www.oie.int), the Food and Agriculture Organization World Reference Laboratory for foot-and-mouth disease (http://www.wrlfmd.org), the Southeast Asia and China Foot-and-Mouth Disease Campaign (http://www.seafmd-rcu.oie.int), national reports for international meetings (Southeast Asia and China Foot-and-Mouth Disease Campaign 2013, World Organisation for Animal Health/Japan Trust Fund on foot-and-mouth disease control in Asia 2013), and previously reported data (*9*–*11*) for Southeast Asia (Vietnam, Cambodia, Myanmar, Thailand, Laos, Malaysia, and the Philippines) and eastern Asia (South Korea, Japan, China, Mongolia, Russia, North Korea, Hong Kong, and Taiwan) regions for 1999–2013. Statistical analysis was performed by using paired or unpaired *t*-tests, and correlation were made by using GraphPad InStat version 3.05 (Graph Pad Software, La Jolla, CA, USA). A phylogenetic tree was inferred by using the neighbor-joining method, and analysis was conducted by using MEGA version 6 (http://www.megasoftware.net/).

**Figure 1:**
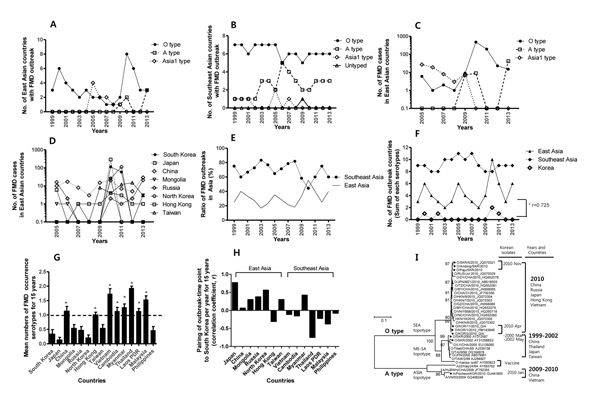
Macro-analysis of foot-and-mouth disease (FMD) outbreaks in countries in eastern and Southeast Asia, 1999–2013. A) Number of countries in eastern Asia (South Korea, Japan, China, Mongolia, Russia, North Korea, Hong Kong, Taiwan) with FMD outbreaks on the basis of serotype. Values indicate sum of duplicated country counts of outbreaks caused by multiple serotypes. B) Number of countries in Southeast Asia (Vietnam, Cambodia, Myanmar, Thailand, Lao People’s Democratic Republic [Laos PDR], Malaysia, and the Philippines) with FMD outbreaks on the basis of serotype. C) Total number of FMD cases in countries in eastern Asia, 2005–2013. D) Number of FMD cases in countries in eastern Asia, 2005–2013, as indicated in the Animal Health Information Database. E) Ratio of FMD outbreaks in countries in eastern and Southeast Asia. F) Number of FMD outbreak countries (sum of FMD outbreak serotypes) in countries in eastern and Southeast Asia. The correlation coefficient (r) for eastern Asia and South Korea was 0.725. G) Mean numbers of FMD virus serotypes detected in countries in Asia and in South Korea during a 15-year period. *p<0.001, by paired *t*-test. Error bars indicate SEM. H) Comparison of numbers of outbreak serotypes of FMD virus in South Korea with those for countries in eastern and Southeast Asia during a 15-year period. The correlation between counties in eastern and Southeast Asia was significant (p<0.05). I) Phylogenetic analysis of viral protein 1 genes of FMD viruses identified in South Korea and countries in Asia during FMD outbreaks, 1999–2013. The phylogenetic tree was inferred by using the neighbor-joining method. Black dots indicate isolates identified in South Korea. Values along the branches are bootstrap values (1,000 replicates). Analysis was conducted by using MEGA version 6 (http://www.megasoftware.net/) and data from the World Organisation for Animal Health (OIE) Information Database (http://www.oie.int), The Food and Agriculture Organization World Reference Laboratory for FMD (http://www.wrlfmd.org), the Southeast Asia and China FMD (SEACFMD) Campaign (http://www.seafmd-rcu.oie.int), national reports for international meetings (SEACFMD 2013,OIE/Japan Trust Fund on FMD control in Asia 2013), and previously reported data (*9*–*11*). Scale bar indicates nucleotide substitutions per site.

Comparative analysis of outbreaks in neighboring countries over the past 15 years showed a high incidence of outbreaks at 2- to 5-year intervals (2000, 2005, 2010–2011, and 2013) (Figure 1, panels A and F). Analysis of outbreak serotypes and cases in neighboring countries (Figure 1, panels A–C) showed that type O foot-and-mouth disease virus has been predominant every year for the past 15 years. Outbreaks in eastern Asia and South Korea over the past 15 years showed a strong relationship with each other (r = 0.725) (Figure 1, panels D–H). Given the overall trend in Asia, serotype Asia 1 was predominant in 2005 (Figure 1, panels A, B). The situation for foot-and-mouth disease in Asia was regarded as serious during 2010–2011 because of the increased numbers of outbreaks (Figure 1, panels A, C–F). In 2013, the number of type A outbreaks increased, and outbreaks caused by types O and A viruses were still considered a threat (Figure 1, panels A, B).

Of 5 outbreaks in South Korea during 2000–2011, 4 were caused by type O virus, 2 were caused by the Middle East–South Asia topotype, and 2 were caused by the Southeast Asia topotype. One of the 5 outbreaks was caused by type A virus (ASIA topotype, Sea-97 lineage) (Table; Figure 1, panel I). Middle East–South Asia topotype viruses that caused outbreaks in 2000 and 2002 were related to PanAsia lineage viruses, which were detected during 1999–2000 in China, Taiwan, Japan, and Thailand; the causative viruses had high genetic similarity (*12*). We assume that these viruses, which have predominated in these regions since 1998 (*12*), were introduced to South Korea in 2000 and 2002. Serotype O viruses that caused outbreaks in 2010 were identified as SEA type, Mya-98 lineage. This virus type was detected in 2010 in Asia, including Russia, Japan, China, Hong Kong, and Vietnam, and the genetic similarity of these viruses was high (Figure 1, panel I). Type A virus (ASIA topotype, Sea-97 lineage) was detected in South Korea in January 2010. This virus is similar to those detected in 2009 in China and Vietnam. Genetic analyses of all viruses detected in South Korea showed a correlation with viruses that predominated in neighboring countries (Figure 1, panel I).

**Table:** Characteristics of 5 outbreaks of foot-and mouth-disease, South Korea, 2000– 2011*

Characteristic	2000 Mar	2002 May	2010 Jan	2010 Apr	2010 Nov– 2011 Apr
Disease status					
No. cases	15	16	7	13	153
No. virus-positive cases	15	16	7	29	3,700
Duration virus detected, d	22	52	28	29	145
Period of virus detection	Mar 24–Apr 15	May 2–Jun 23	Jan 2–Jan 29	Apr 8–May 6	Nov 28–2011 Apr 21
Host tropism	Ruminant	Pig (cattle)	Ruminant	Ruminant, pig	Ruminant, pig
Serotype (topotype/lineage)	O (ME-SA/PanAsia)	O (ME-SA/PanAsia)	A (ASIA/SEA-97)	O (SEA/Mya-98)	O (SEA/Mya-98)
No. affected provinces (cities or counties)	3 (6)	2 (4)	1 (2)	4 (4)	11 (75)
Economic losses, US$, millions	300	143	29	124	3,000
Date of disease-free status	2001 Sep 16	2002 Nov 29	2010 Sep 27	2010 Sep 27	2014 May 29
Control measures					
Eradication policy	Culling, vaccination	Culling	Culling	Culling	Culling, vaccination
No. cattle culled	2,021	1,372	2,905	10,858	150,864
No. pigs culled	63	158,708	2,953	38,274	3,318,298
No. other animals culled	132	75	98	742	10,800
Total culled	2,216	160,155	5,956	49,874	3,479,962
Area of culling, km radius	0.5 (all)	0.5 (all), 3 (pigs)	0.5	0.5, 3 (on 2 farms)	0.5
Vaccine strain	O Manisa	NA	NA	NA	O Manisa
No. animals vaccinated	1st: 860,700, booster: 661,700	NA	NA	NA	All susceptible animals
Vaccination range, km radius	10	NA	NA	NA	Nationwide
Serosurveillance area, km radius	20	10	10	10	10
Restricted zones, km radius					
Management	NA	NA	10–20	10–20	10–20
Surveillance	10–20	3–10	3–10	3–10	3–10
Protection	0–10	0–3	0–3	0–3	0–3
Putative sources					
Regions in Asia as possible sources	Northeastern	Northeastern	Northeastern	Northeastern	Southeastern
Major sources of first outbreak	International travelers, imported hay	Overseas travel, foreign workers	Foreign workers, international parcels	Overseas travel	Overseas travel
Low possibility sources of first outbreak	Windborne spread of contaminated yellow sand, wild birds	Swill, saw dust, wild animals and birds, yellow sand	Overseas travel, imported forage, TMR feed, saw dust	Imported forage, TMR feed	Foreign workers, illegal livestock products
Possible transmission factor for domestic regions	Imported hay	Humans and vehicles	Humans (veterinarians, meetings, animal feeding)	Vehicles, humans	Vehicles, humans
References†	(*2,8*)	(*3*)	(*4*)	(*6*)	(*5,7*)

*ME-SA, Middle East–South Asia; USD, US dollars; TMR, total mixed ration; NA, not applicable. †Data were obtained from national epidemiology investigation reports on the 5 foot-and-mouth disease outbreaks in South Korea and the references.

**Figure 2:**
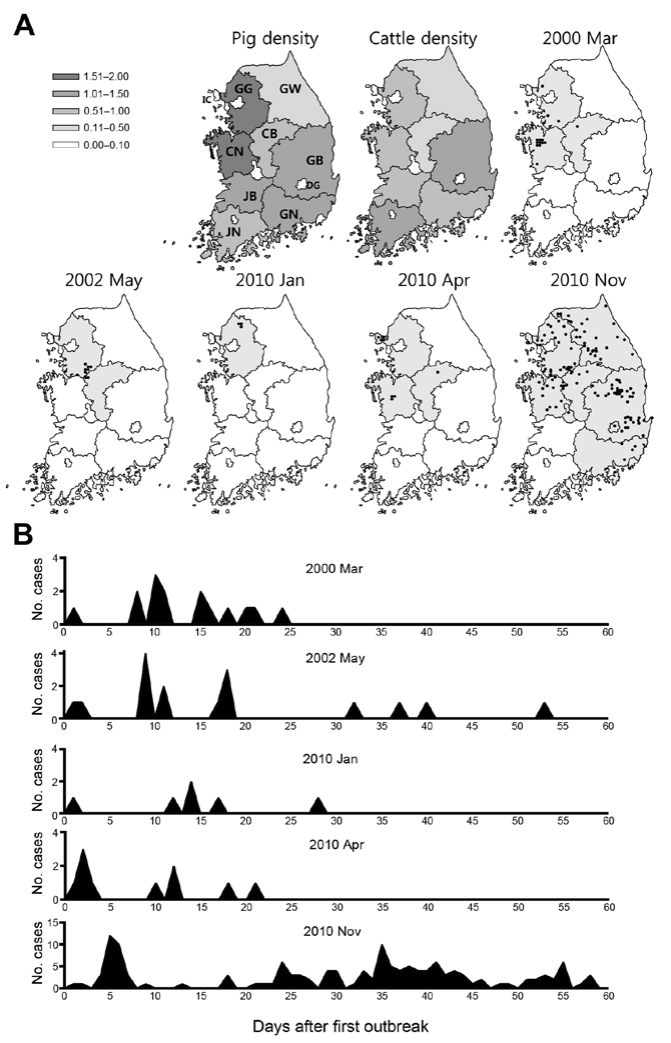
Affected regions and detection time points during 5 foot-and-mouth disease outbreaks, South Korea, 2000–2010. A) Affected provinces and regions and the densities of livestock (pigs and cattle) in 2010 on the basis of data from the Korean Statistical Information Service (http://www.kosis.kr). Values in the key are in millions. IC, Incheon; GG, Gyeonggi; GW, Gangwon; CN, Chungnam; CB, Chungbuk; GB, Gyeongbuk; GN, Gyeongnam; JN, Jeonnam; JB, Jeonbuk; DG, Daegu. B) Comparison of detection time points of cases of foot-and-mouth disease.

Major putative factors for inter-regional or inter-farm virus transmission during the 5 foot-and-mouth disease outbreaks in South Korea were movement of humans or vehicles (Table). The 5 outbreaks that occurred since 2000 were analyzed by province (Figure 2, panel A). The disease occurred most frequently in Gyeonggi Province (5 times), followed by Chungbuk Province (4 times) and Chungnam Province (3 times). Therefore, these 3 provinces, which had the highest risk for infection, were characterized by a high density of pig and cattle farms. On the basis of analysis of 4 outbreaks, the second round of outbreaks occurred 8.0 ± 2.0 days after the first infected group had been identified (Figure 2, panel B). In the most recent outbreak in November 2010, the initial diagnosis was delayed for 1 week; many concurrent infections were detected, and no unique aspects of transmission after the first detection of the disease had been identified (Figure 2, panel B). Most infections occurred ≤25 days after the initial case, after which occurrence was intermittent (Figure 2, panel B).

## Conclusions

International trade and globalization have recently been indicated as major factors for transmission of infectious diseases associated with livestock (*6*). Multiple sources of serotypes O, A, and Asia 1 of foot-and-mouth disease viruses, which have caused recent outbreaks in eastern Asia, are endemic to Southeast Asia (*13*). Incursion of these viruses from Southeast Asia into eastern Asia has been suggested because of the porous nature of borders (*13*). However, the Korean Peninsula is surrounded by water on 3 sides and shares its only land border with North Korea. We believe that inflow of illegal live animals and livestock products, which is generally the highest risk factor for foot-and-mouth disease (*14*), is negligible in the regions around South Korea. Access to suspected infectious materials from countries with outbreaks is fundamentally blocked by shipping regulations.

Although no evidence for confirmation of introduction is available, results of epidemiologic investigations have indicated that the 5 foot-and-mouth disease outbreaks in South Korea were related primarily to indirect transmission by humans who came into contact with suspected infectious animals or livestock products from countries in Asia to which the virus is endemic (Table) (*2*–*7*). In addition, imported hay or other imported animal products were probable sources of virus in March 2000 and January 2010 (Table) (*2*,*4*,*8*), and the viruses were transmitted to persons who had contact with these materials directly or indirectly. On the basis of the national mandatory reporting system for foreign workers (http://www.kahis.go.kr), we found that the number of persons from Vietnam, Cambodia, Thailand, and China who work on farms in South Korea has been increasing since 2005. The recent situation can be regarded as conducive for an increased risk for foot-and-mouth disease.

The outbreak pattern of foot-and-mouth disease in South Korea was more strongly correlated with outbreaks in countries in eastern Asia than with outbreaks in Southeast Asia. Outbreaks every 15 years caused by type O foot-and-mouth disease virus are predominant in Asia. The greatest risk for infection is currently by type O and A viruses, followed by type Asia 1 virus.

In summary, type O foot-and-mouth disease virus was responsible for 4 outbreaks in South Korea and type A virus accounted for 1 outbreak. South Korea might be at risk for foot-and mouth disease, given the high incidence of this disease at 2- to 5-year intervals (2000, 2005, 2010–2011, and 2013) in eastern Asia. Foot-and-mouth disease outbreaks in neighboring countries were a probable major source of introduction of this disease into South Korea. Once this disease is introduced, prevention of domestic transmission should include extensive restriction of movement of humans or vehicles during an outbreak.
